# Effect of the Uniaxial Compression on the GaAs Nanowire Solar Cell

**DOI:** 10.3390/mi11060581

**Published:** 2020-06-10

**Authors:** Prokhor A. Alekseev, Vladislav A. Sharov, Bogdan R. Borodin, Mikhail S. Dunaevskiy, Rodion R. Reznik, George E. Cirlin

**Affiliations:** 1 Ioffe Institute, 194021 Saint-Petersburg, Russia; vl_sharov@mail.ru (V.A.S.); brborodin@gmail.com (B.R.B.); mike.dunaeffsky@mail.ioffe.ru (M.S.D.); 2 Alferov University, 194021 Saint-Petersburg, Russia; cirlin.beam@mail.ioffe.ru; 3 ITMO University, 197101 Saint-Petersburg, Russia; moment92@mail.ru; 4 Saint Petersburg Electrotechnical University “LETI”, 197376 Saint-Petersburg, Russia

**Keywords:** GaAs, gallium arsenide, nanowire, solar cell, piezoelectric, polarization, piezophototronic, piezoresistance, zinc blende, wurtzite

## Abstract

Research regarding ways to increase solar cell efficiency is in high demand. Mechanical deformation of a nanowire (NW) solar cell can improve its efficiency. Here, the effect of uniaxial compression on GaAs nanowire solar cells was studied via conductive atomic force microscopy (C-AFM) supported by numerical simulation. C-AFM I–V curves were measured for wurtzite p-GaAs NW grown on p-Si substrate. Numerical simulations were performed considering piezoresistance and piezoelectric effects. Solar cell efficiency reduction of 50% under a −0.5% strain was observed. The analysis demonstrated the presence of an additional fixed electrical charge at the NW/substrate interface, which was induced due to mismatch between the crystal lattices, thereby affecting the efficiency. Additionally, numerical simulations regarding the p-n GaAs NW solar cell under uniaxial compression were performed, showing that solar efficiency could be controlled by mechanical deformation and configuration of the wurtzite and zinc blende p-n segments in the NW. The relative solar efficiency was shown to be increased by 6.3% under −0.75% uniaxial compression. These findings demonstrate a way to increase efficiency of GaAs NW-based solar cells via uniaxial mechanical compression.

## 1. Introduction

GaAs nanowire (NW) is a prospective material for third-generation solar cells [1,2,3]. GaAs planar solar cells exhibit high efficiency [4], but they are less cost-effective than Si-based solar cells [5]. Modern technology allows the growth of GaAs NW on low-cost substrates [6] to engineer solar light absorption by varying geometrical size and spacing of NWs [7,8,9,10,11]. Formation of an array of NWs reduces material consumption during growth with respect to conventional planar growth. Recently, GaAs NW solar cells with 15.1% efficiency were developed [12], where the solar cells were grown on the GaAs substrate. To improve solar cell efficiency, additional approaches are required. Formation of tandem solar cell GaAs nanowire/silicon substrates [13,14,15] and exploitation of the piezophototronic effect are among these approaches currently being investigated.

The piezophototronic effect proposed by Z.L. Wang couples electric fields induced by piezoelectric polarization with photogenerated charge carriers [16,17]. The piezoelectric field enhances separation of photogenerated carriers and boosts efficiency of the solar cell. Growth of III–V NWs occurs along the (111) direction, whereby III–V materials with zinc blende (ZB) structures possess non-zero piezoelectric constants [18] and deformation along the (111) direction induces collinear piezoelectric polarization [19]. For InP/InAs axial and radial heterostructures, lattice mismatch between the heterolayers was shown to lead to piezoelectric polarization along the (111) direction [20], a phenomenon that can be exploited in solar cells [21]. For GaAs (111) diodes, external deformation was shown to shift the Schottky barrier height [22]. Recently, highly sensitive piezotronics pressure sensors were developed based on undoped zinc blende GaAs NWs and demonstrated a high piezotronic sensitivity to pressure of ~7800 meV/MPa [23]. The growth of zinc blende or wurtzite GaAs NWs on Si substrates would also be assumed to induce piezoelectric polarization due to the strain created by the difference between the GaAs and Si lattice constants [24], but the impact of such polarization on the electrical properties of GaAs/Si solar cells has not yet been studied.

Growth of III–V NWs can be conducted with wurtzite (WZ) crystal structures, in which piezoelectric constants are increased with respect to zinc blende structures [18,25]. Enhancement of solar cell efficiency due to the piezophototronic effect was demonstrated for ZnO [26], CdS [27] and III-N [28] nanowires with wurtzite crystal structures. AlN, GaN and ZnO piezoelectric constants are higher than in GaAs [25]. Additionally, these materials have wide band gaps; therefore, piezoelectric polarization governs their electronic properties under deformation. For wurtzite GaAs NWs, compression of 1% along the growth axis reduces the band gap by 0.1 eV and increases electron affinity by 0.15 eV [29], which should be considered when studying deformation-induced effects.

The impact of mechanical deformation on GaAs nanowire-based solar cells with wurtzite structures has not yet been studied. The aim of this work was to study wurtzite GaAs NW/p-Si solar cell efficiency with uniaxial compression of GaAs NWs. Additionally, the impact of the compression on GaAs NW-based solar cells with various configurations of WZ and ZB segments was calculated.

## 2. Materials and Methods

**Figure 1:**
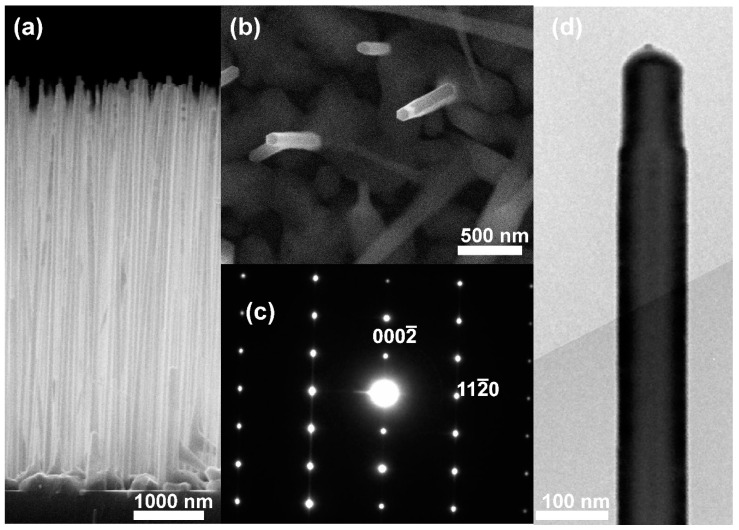
GaAs nanowires. (**a**) Side view and (**b**) top view SEM images of the GaAs nanowires array grown on Si substrate; (**c**) electron diffraction pattern of the single nanowire (NW); (**d**) TEM image of single nanowire.

GaAs nanowires were grown by molecular beam epitaxy on a p-Si (111) substrate with a doping level of 10^16^ cm^−3^ using catalytical Au nanoparticles with a diameter of 20 nm. The p-type doping level (Be) was in the range of 10^17^–10^18^ cm^−3^. To reduce the surface state density and increase solar efficiency, NWs were passivated using a 7-nm coating layer of AlGaAs, with an NW length L = 6 μm and a diameter d = 100 ± 20 nm [30]. After the growth of GaAs core, the substrate temperature was decreased to ensure layer by layer growth mechanism of AlGaAs shell. This mechanism implies absence of an AlGaAs layer under Au cap. According to previous growth rate calibrations the nominal Al content in Al_x_Ga_1−x_As shell was x = 0.3. To promote NWs growth, we have used calibrated Au nanoparticles with average diameter of 20 nm. These particles were randomly dispersed onto substrate and subjected to a special procedure described in [31]. The procedure of Au nanoparticles deposition, in principal, allows one to control the density of the seeds. In our particular case, the mean distance between the particles was set at 500 nm. Mean separation distance was confirmed by a top view scanning electron microscopy (SEM) image of the nanowire array (see Figure 1b). Surface density of NWs was of ~4 × 10^8^ cm^−2^. The chosen separation between the NWs is suitable for AFM manipulation with single NW. Figure 1a,b shows SEM images of the nanowire array, with the NWs exhibiting wurtzite crystal structures. Figure 1c,d shows electron diffraction pattern and the transmission electron microscopy (TEM) image of an NW. Now it is well documented that a contact angle of the droplet dictates the crystal phase of the NWs [32]. In common case, if the angle is smaller than 120° the WZ phase dominates. This value of angle is achieved by low-temperature growth under As-rich conditions. These are exactly conditions we have used during the growth. Conventionally, the growth of the wurtzite GaAs nanowires on the Si substrate started from zinc blende phase [33]. Zinc blende stacks ~10 nm thick were anticipated at the bottom of the NWs.

**Figure 2:**
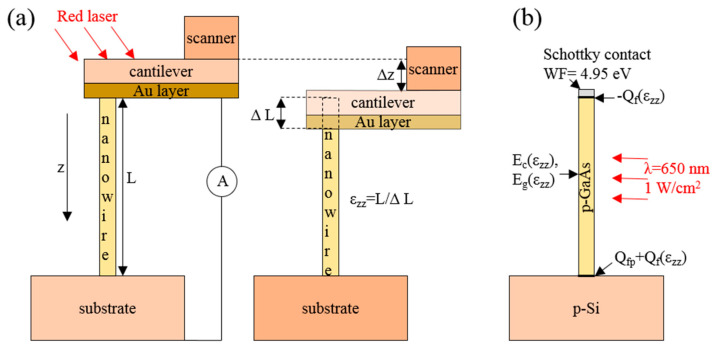
(**a**) Scheme of the measurement of the GaAs NW I–V curve by atomic force microscopy (AFM) cantilever (left) and applying of the compression strain ε_zz_ to the NW by the cantilever (right); (**b**) scheme of the positioning of the parameters implemented in the calculated model.

To study the effect of deformation of a single nanowire on solar cell efficiency, conductive atomic force microscopy (C-AFM) experiments were performed [34,35]. An NTegra AURA microscope (NT-MDT) was used for these measurements, where HA_NC/W2C + (NT-MDT) probes with conductive W_2_C coatings and a cantilever stiffness value k_c_ = 15 N/m were used. The back side of the cantilever was coated with Au by a manufacturer. To achieve compression deformation of the GaAs NWs, contact between the probe and the NW top was required to be stable, with top of the NW pinned at the probe surface. For this purpose, a chip with the cantilever was placed upside down and the Au coating of the cantilever made contact with NW top by moving the probe vertically. Compression of the NW was performed by further moving the probe vertically (see Figure 2a). Vertical movement of the probe was performed using a step of Δz = 200 nm. At each step, I–V curves of the NW were measured via C-AFM and a built-in amperemeter with a sensitivity of ~1 pA. The probe was grounded during measurements and bias voltage was applied to the silicon substrate. Since cantilever deflection was registered by a red laser, NWs were illuminated by a monochromatic radiation with a power density of ~10^4^ W/m^2^ and with a wavelength of ~650 nm. To ensure contact of the cantilever with a single NW, neighboring NWs were broken with the probe by scanning in a contact mode before measuring the I–V curves. Details of a procedure are presented in Figure S1 (see Supplementary Material).

The NW strain ε_zz_ in the vertical direction was estimated by the expression ε_zz_ = −ΔL/L, where L and ΔL represent the NW length and a change in NW length, respectively. To estimate the ε_zz_ induced by vertical movement of the probe, the stiffness of the cantilever needed to be considered. The strain can be estimated by expression: ε_zz_ = k_c_ × Δz/(L × ((π × d^2^ × E/4L) + k_c_)), where k_c_—stiffness of the cantilever, Δz—vertical movement of the probe, d—NW diameter, L—NW length, E—Young’s modulus. For the wurtzite GaAs with Young’s modulus (E = 141 GPa) [36], the values of the obtained strain in the (111) direction along an NW with a diameter of 100 nm were ε_zz_ ≈ −0.25% for Δz = 200 nm and ε_zz_ ≈ −0.5% for Δz = 400 nm. These strain values were used to model the experimental I–V curve, assuming that the strain was uniformly distributed along the NW. In our experimental geometry, moving the probe more than Δz = 400 nm would have induced strains higher than −0.5% with simultaneous buckling of the NW [37,38], in which the strain would have been nonuniformly distributed and analysis of the experimental data would have been much more complicated. Typical “force-distance” loading curve was linear for Δz up to ~400 nm (see Figure S2 in Supplementary Material), indicating absence of buckling during an I–V curves measurement.

**Figure 3:**
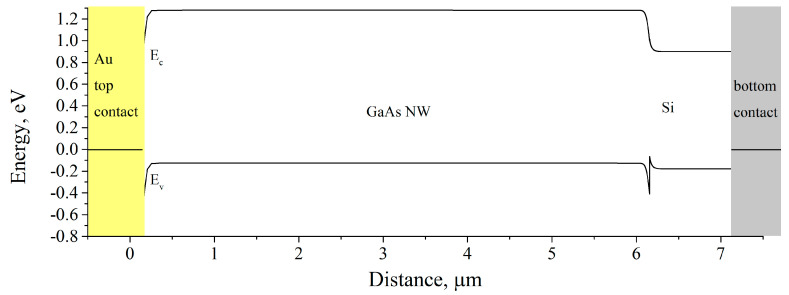
Band diagram of the grounded Au/p-GaAs/p-Si heterostructure in dark conditions.

Modeling of the I–V curves under red laser illumination was performed in 2D with cylindrical symmetry using the Atlas Silvaco software package [39]. The model replicated cylindrical GaAs with a diameter of 100 nm and a length of 6 μm placed on the 1 μm thick and 10 μm wide Si substrate. Affinity of the Si substrate was 4.05 eV. Doping of the substrate was p-type with a level of 10^16^ cm^−3^. P-type doping of the NW was varied during calculations to achieve the best fit. The bottom contact between the Si substrate and a sample holder was set as Ohmic and the top contact with the NW was a Schottky-type, assumed to cover the top plane of the NW. The top contact was assumed to be Au/GaAs since AlGaAs was absent below Au cap. Schottky barrier height of Au/GaAs NW contact was of 0.54 eV [40]. Band diagram of the grounded structure in dark conditions is presented in Figure 3. Thermionic emission, recombination and tunneling across the Schottky barrier were calculated using the built-in universal Schottky tunneling model. The thermionic emission current was calculated according to the surface recombination velocity and band-to-band recombination. The hole mobility was reduced to 0.1 cm^2^/(V·s) due to a strained GaAs/Si interface and possible planar defects in the NW [41].

Red laser illumination in a model (monochromatic with a wavelength of ~650 nm and a power density of 10^4^ W/m^2^) was considered by implementing an optoelectronic generation/recombination process, whereby the illumination was set with uniform space distribution and normal incident on the side surface. Enhancement of the light absorption due to the NW and array geometry were not considered.

The impact of mechanical deformation on the NW I–V curves was introduced by influencing both the piezoelectric and piezoresistance effects. Since the piezoelectric effect led to the formation of the opposite charges at the top and bottom planes of the NW, fixed charges, i.e., Q_f_ and −Q_f_, were set at the GaAs/Si and GaAs/top contacts, respectively (Figure 2b). The positive charge was always at the GaAs/Si interface because it was induced at the bottom of the GaAs NW grown in the 111B direction under compression [23]. The Q_f_ charges per 1% strain for the zinc blende and wurtzite GaAs are presented in Table 1. The density of the fixed charge was calculated according to the expression Q_f_ = e_ij_ε_zz_/e, where e_ij_ represents the piezoelectric constants (e_14_ = −0.16 C/m^2^ or e_33_ = −0.295 C/m^2^ the for zinc blende [18] and wurtzite GaAs [25,30], respectively) and e represents the elementary charge (1.6 × 10^−19^ C). The piezoresistance effect was calculated by introducing changes in the conduction band minimum (electron affinity) E_c_ and the band gap E_g_ under compression into the model, of which the values were taken from references [42] and [29] for the zinc blende and wurtzite GaAs, respectively, and are presented in Table 1. E_c_ for unstrained WZ and ZB GaAs were assumed to be 4.07 eV. Changes in Q_f_, E_c_ and E_g_ were assumed to be linear with regard to the strain ε_zz_. Under a compression strain a E_c_ in WZ GaAs is increased, while E_c_ in ZB GaAs is decreased due to opposite behavior of conduction band minima Γ_8_ and Γ_6_ in WZ and ZB GaAs, respectively.

Conventionally, a Schottky barrier height for n-type semiconductor (Φ_bn_) can be estimated by a Schottky-Mott rule: Φ_bn_ = Φ_m_ − E_c_, where Φ_m_ – metal work function, E_c_ – electron affinity of a semiconductor. This rule is not valid for semiconductors with a high density of interface electronic states. In GaAs with a high density of surface or interface electronic states, a Schottky barrier height is not following to the Schottky-Mott rule [43]. For this case, an effective work function (EWF) model can be used [44]. According to this model, a Schottky barrier height can be estimated by an expression Φ_bn_ = Φ_eff_ − E_c_, where Φ_eff_—effective work function. For p-type semiconductor Φ_bp_ = (E_g_/e) − Φ_eff_ + E_c_. Notably, the EWF model already takes into account the density of interface states, since the effective work function is determined by the position of pinning of the Fermi level at these states. Recently, it was shown that the Fermi level in III-As semiconductors was pinned due to the formation of excess surface arsenic at the oxidized or metalized surface [45]. The position of pinning (effective work function) was ~4.8 eV for n-type and ~4.95 eV for p-type semiconductors, implying insensitivity to mechanical deformation [46,47]. During modeling, the work function of the contact was set at Φ_eff n_ = 4.8 and Φ_eff p_ = 4.95 eV for n-type and p-type GaAs, respectively.

## 3. Results and Discussion

This section firstly presents the experimental and theoretical results of the WZ p-GaAs NWs on p-Si substrates, followed by the results of the numerical modeling of the p-n GaAs nanowire solar cells with different combinations of WZ and ZB segments under uniaxial compression.

### 3.1. WZ p-GaAs NW Grown on p-Si Substrate

**Figure 4:**
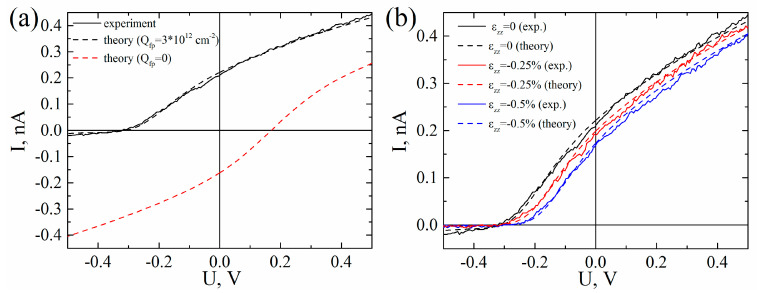
I–V curves of the p-GaAs NW grown on p-Si substrate. Voltage axis shows a voltage applied to the p-Si substrate with respect to the top of the NW. (**a**) Measured curve of the unstrained NW (black solid) and calculated curves with a fixed charge Q_fp_ at the GaAs/ Si interface (black dashed) and without fixed charge (red dashed curve). (**b**) Experimental (solid curves) and calculated (dashed) I–V curves obtained for the uniaxial compression strain ε_zz_ = 0%, −0.25%, −0.5%, (black, red and blue curves, respectively).

Figure 4a shows the I–V curve (black solid) measured by C-AFM on the unstrained GaAs NWs. During measurement, bias voltage was applied to the p-Si substrate and a negative open-circuit voltage (V_oc_) in the I–V curve were observed. The red dashed line in Figure 4a represents a calculated I–V curve for the NWs, with a p-type doping level of 10^17^ cm^−3^. The V_oc_ in the calculated curve was positive, with the polarity of the V_oc_ remaining positive during p-type doping with a level of 10^18^ cm^−3^ and for NW with various levels of n-type doping (not shown here). The charge of V_oc_ was governed by the polarity of the photogenerated current, which was in turn controlled by the balance between the barriers at the NW/top contact and NW/substrate interfaces. In our case, the Schottky barrier presented a bigger impact with respect to the GaAs/Si heterobarrier. For accurate modeling, a charge induced by the lattice constant mismatch between the Si and the GaAs was required. Lattice mismatch in nitrides with wurtzite crystal structures is known to induce charges at the interface [48]. A similar process occurs for ZB layers grown in the (111) direction [19]. During growth, strain caused by the mismatch in the lattice constants of GaAs and Si (~4%) was relaxed at the NW surface decreased along the NWs, leading to the gradual increasing of the lattice constant along the NWs [24]. In III-N structures, this process induced a negative fixed charge near the interface [49]. The sign of the e_33_ constant in the III-N structures was positive, but in GaAs the e_33_ was negative [25]; therefore, a positive fixed charge Q_fp_ was anticipated. The exact volume of the charge was hard to estimate due to the unknown thickness of the ZB segment and a complex distribution of strain at the nanowire base. During modeling, Q_fp_ and p-type doping level N_a_ values were varied to obtain the best fit according to the experimental I–V curve. The red dashed line in Figure 4a represents the N_a_ = 10^17^ cm^-3^ and Q_fp_ = 3 × 10^12^ cm^−2^ calculations occurring at the NW/substrate interface.

Figure 4b shows the I–V curves obtained on the unstrained (black solid) and compressed GaAs NWs after vertical movement of the cantilever at Δz = 200 nm (red solid curve) and Δz = 400 nm (blue solid curve). The dashed lines correspond to the calculated curves. Calculations were performed assuming that ε_zz_ ≈ −0.25% for Δz = 200 nm and ε_zz_ ≈ −0.5% for Δz = 400, with Q_f_ being added to the Q_fp_ at the NW base. The modeled I–V curves satisfactorily coincided with the experiment. Figure 4b shows that compression of the p-GaAs NWs led to decreased efficiency of the WZ p-GaAs NW/p-Si solar cells by 40%.

**Figure 5:**
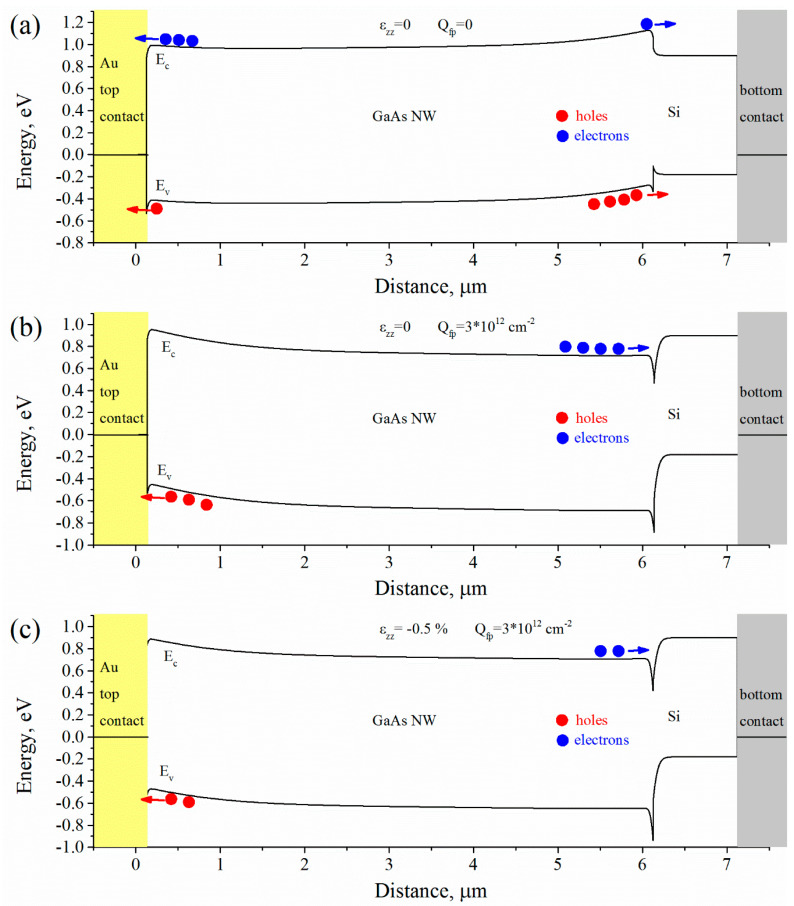
Band diagrams calculated for WZ p-GaAs NW grown on p-Si substrate illuminated by a monochromatic radiation at short circuit conditions. Electrons and holes are shown in blue and red circles, respectively. (**a**) Unstrained NW without fixed charge at the GaAs/Si interface; (**b**) unstrained NW with fixed charge at the GaAs/Si interface Q_fp_ = 3 × 10^12^ cm^−2^; (**c**) Uniaxially strained (ε_zz_ = −0.5%) NW with fixed charge at the GaAs/Si interface Q_fp_ = 3 × 10^12^ cm^−2^.

**Figure 6:**
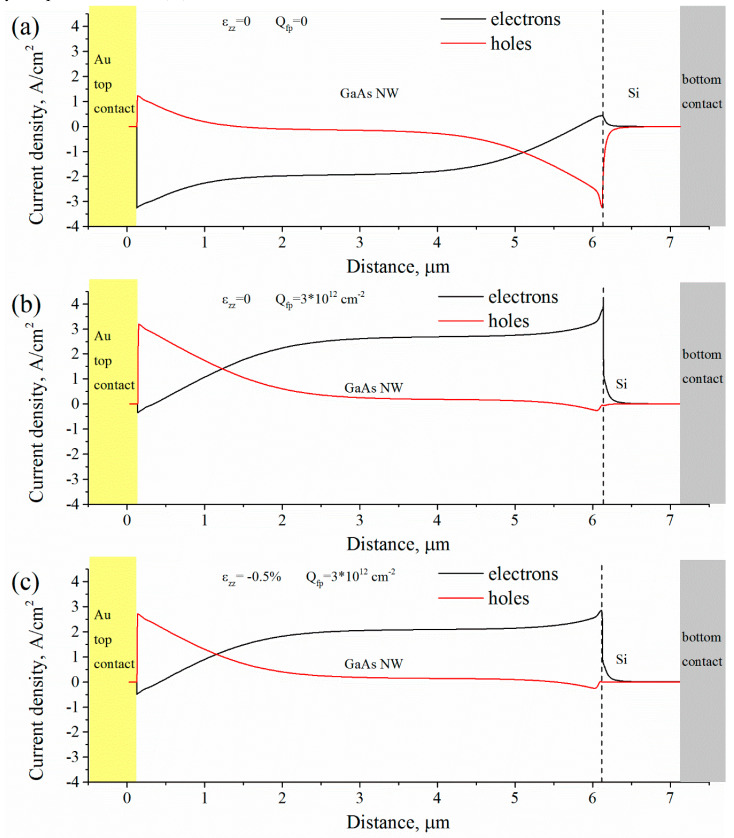
Distribution of electronic (black curves) and hole (red curves) photocurrent densities along Au/p-GaAs/p-Si heterostructure illuminated by a monochromatic radiation at short circuit conditions. (**a**) Unstrained NW without fixed charge at the GaAs/Si interface; (**b**) unstrained NW with fixed charge at the GaAs/Si interface Q_fp_ = 3 × 10^12^ cm^−2^; (**c**) uniaxially strained (ε_zz_ = −0.5%) NW with fixed charge at the GaAs/Si interface Q_fp_ = 3 × 10^12^ cm^−2^.

Figure 5 shows band diagrams calculated for WZ p-GaAs NW grown on p-Si substrate illuminated by a monochromatic radiation with a power density of ~ 10^4^ W/m^2^ and with a wavelength of ~650 nm at short circuit conditions. Band diagrams in Figure 5a–c are corresponding to red, black (in Figure 4a) and blue (in Figure 4b) I–V curves, respectively. Profiles of corresponding electronic and hole photocurrent densities along a structure are presented in Figure 6. From Figure 5a it follows that Au/GaAs and GaAs/Si barriers are connected in opposite directions. However, at the Au/GaAs barrier an electronic photocurrent dominates a hole photocurrent and at the GaAs/Si barrier a hole photocurrent dominates an electronic photocurrent (see Figure 6a). This leads to a negative I_sc_ and positive V_oc_ in the I–V curve Introducing of a fixed charge Q_fp_ induced by a lattice mismatch at the GaAs/Si interface changes balance of the Au/GaAs and GaAs/Si barriers (see Figure 5b and Figure 6b). In this case electronic and holes photocurrents change their directions and I_sc_ become negative with positive V_oc_. Adding of piezoelectric charge Q_f_ at Au/GaAs and GaAs/Si interfaces unbends bands (Figure 5c) near the barriers with simultaneous decreasing of the I_sc_ (Figure 6c).

Contributions of a piezoresistance and piezoelectric effects to the solar efficiency were also separated during a modeling. I_sc_ and V_oc_ were calculated for the ε_zz_ ≈ −0.5%, assuming Q_f_ = 0. Thus, only impact of the piezoresistance and a Q_fp_ (charge induced by the lattice constant mismatch at the GaAs/Si interface) were accounted. Switching off the piezoelectric effect lead to a simultaneous decreasing of the I_sc_ and V_oc_ by 0.2% and 2.2% respectively. That gives a negligible changing of the solar efficiency. Negligible impact of the piezoelectric effect on the solar efficiency in our case can be explained by a relatively low Q_f_ with respect to Q_fp_ (~3 times lower) and a high doping level of a NW. High doping and a light illumination reduce piezopotential induced by a Q_f_ at a Schottky barrier region. Changing of the Schottky barrier height by the Q_f_ is negligible with respect to a contribution by the piezoresistance (E_c_).

Thus, growth of the GaAs NWs on the (111) Si substrate induced a positive fixed charge at the GaAs/Si interface arising from the crystal lattice mismatch at the interface. The impact of the charge led to the changing of the V_oc_ sign in the solar cell, an important effect previously unconsidered in known publications but which must be regarded when designing high-efficiency tandem GaAs NW/Si solar cells. Uniaxial compression of the WZ p-GaAs NW on the p-Si substrate reduced solar cell efficiency by 50% at a strain of −0.5%. However, compression of the GaAs NWs may lead to increased efficiency in structures with various p- and n-layer compositions.

### 3.2. Axial p-n GaAs NW Solar Cell

**Figure 7:**
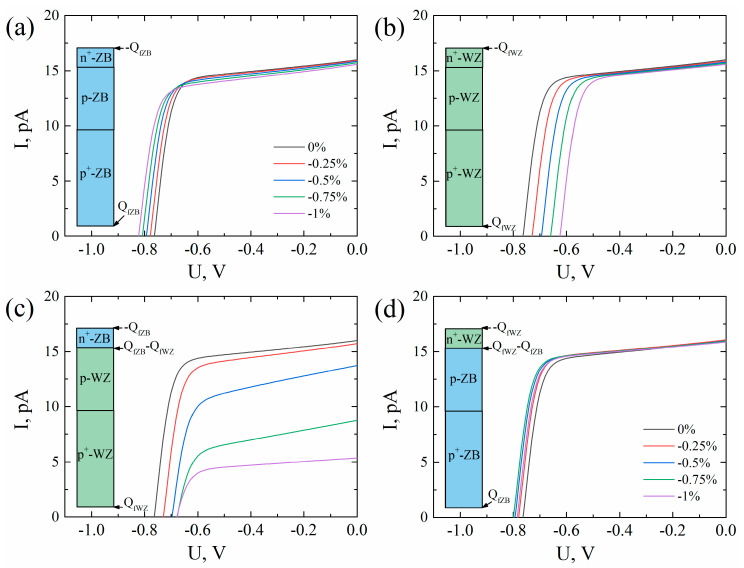
Calculated I–V curves of the GaAs nanowires with different crystal structure under compression strain along NW. Black, red, blue, green and magenta curves correspond to the strain of 0%, −0.25%, −0.5%, −0.75%, −1%, respectively. Insets illustrate crystal structure of the NW and a position and a sign of the polarization charge. (**a**) pure ZB p-n NW; (**b**) pure WZ p-n NW; (**c**) n-ZB/p-WZ NW; (**d**) n-WZ/p-ZB NW.

To find a possible GaAs nanowire structure whereby uniaxial compression increased light conversion efficiency, we performed modeling using the solar cell presented in [12] as a reference. The model replicated the geometry (diameter 185 nm, length 3 μm), doping level (p^+^ 10^18^ cm^−3^–p 10^17^ cm^−3^–n^+^ 3 × 10^18^ cm^−3^) and surface recombination velocities. Lengths of the p^+^, p and n^+^ parts were 1.8 μm, 1 μm and 0.2 μm, respectively. The bottom contact (at the p^+^-side) was set as Ohmic and the top contact (at n^+^-side) was set as Schottky with a work function of 4.8 eV. Four types of possible structures were modeled, namely, pure ZB NW, pure WZ NW, ZB n^+^-segment/WZ p-segment and ZB p-segment/WZ n-segment (see Figure 7). Compression of the NW was calculated by applying the parameters presented in Table 1 to each segment of the NW. Positive charges were placed closer to the NW base and negative charges closer to the top of the NW. Light was modeled as AM 1.5 radiation with normal incidence on the side surface of the NW.

Figure 7 shows the I–V curves calculated for four configurations of the WZ/ZB segments in an NW under different compression strain values ε_zz_. In each configuration, I–V curves were calculated according to ε_zz_ from 0% to −1% in steps of −0.25%. Changes in the NW solar cell efficiency for different configurations with respect to the unstrained ZB NWs are presented in Table 2. Notably, −1% compression increased the relative efficiency of the pure ZB NW by 2.3%, but reduced efficiency of the pure WZ NW by 21.3%. Such opposite efficiency behaviors were mainly due to similar opposite behaviors of the E_c_ in the WZ and ZB GaAs under strain (see Table 1). This finding shows that both piezoresistance and piezoelectrical effects must be considered when analyzing strain-induced effects in GaAs. Adding WZ segments to ZB NWs dramatically reduced the efficiency by 73% with respect to p-WZ/n-ZB NWs under compression. The relative efficiency of the n-WZ/p-ZB configuration was nonmonotonically changed with the strain, reaching 6.3% at a strain of −0.75%.

The presented results showed that mechanical deformation of the GaAs NW controlled efficiency of the NW-based solar cells. Conventionally, in an electric circuit describing electrical processes in NWs, two diodes exist connected back-to-back, with the first diode being a Schottky diode and the second a diode describing a p-n junction or a heterojunction [50]. The shape of an I–V curve is governed by the balance between these two diodes. Numerical modeling allows for the quantitative calculation of I–V curves in complex systems. For the first time, both piezoresistance and piezoelectric effects were considered in the calculations from this work. The contribution of piezoresistance is governed by the relationships between E_c_, E_g_ and the work function of the electrode. Conventionally, a Schottky barrier is formed between GaAs and deposited contacts [45]; however, a high doping level [51,52] or insertion of additional heterolayers can reduce or eliminate this barrier, thereby reducing the impact of the piezoresistance. The calculations in this work were performed under the assumption that the NWs were grown in the (111) B direction; however, they can also be grown in the (111)A direction [53], in which the polarity of the piezoelectric charges would be opposite. Moreover, a thick passivation shell would significantly change the band gap of the NWs [54]. These effects should be considered in future studies.

## 4. Conclusions

To conclude, effects of uniaxial compression on GaAs nanowire solar cells were studied. Wurtzite p-GaAs NWs grown on p-Si substrate were studied by C-AFM. Uniaxial compression along NW growth direction was applied using an AFM probe, which simultaneously acted as a top electrode. Light conversion efficiency was studied by analyzing the measured I*–*V curves and numerical simulations considering both piezoresistance and piezoelectric effects demonstrated agreement with an experiment. Moreover, the analysis showed the presence of a fixed electrical charge at the NW/substrate interface, which was induced due to the mismatch of the crystal lattices and significantly affected solar cell efficiency. Additionally, numerical simulations of the p-n GaAs NW solar cells under uniaxial compression were performed, showing that solar efficiency could be controlled by mechanical deformation and configuration of the WZ and ZB p-n segments in the NWs. The relative solar efficiency was shown to be increased by 6.3% under −0.75% uniaxial compression.

## Figures and Tables

**Table 1:** Parameters of the Q_f_, E_c_ and E_g_ for unstrained and strained with ε_zz_ = −1% zinc blende (ZB) and wurtzite (WZ) NWs.

Structure	Q_f_ (ε_zz_ = 0)	E_c_ (ε_zz_ = 0)	E_g_ (ε_zz_ = 0)	Q_f_ (ε_zz_ = −1%)	E_c_ (ε_zz_ = −1%)	E_g_ (ε_zz_ = −1%)
ZB	0	4.07 eV	1.42 eV	1 × 10^12^ cm^−2^	4.01 eV	1.52 eV
WZ	0	4.07 eV	1.42 eV	1.8 × 10^12^ cm^−2^	4.22 eV	1.32 eV

**Table 2:** Relative changing of the solar cell efficiency of GaAs NW with various crystal structures with respect to p-n NW with pure ZB structure.

ε_zz_,%	Pure p-n ZB, %	Pure p-n WZ, %	n-WZ/p-ZB, %	p-WZ/n-ZB, %
−0.25	1.2	−5.4	3.7	−9.2
−0.5	1.8	−10.8	5.2	−32.6
−0.75	2.1	−16.3	6.3	−61.9
−1	2.3	−21.3	4.0	−73.0

## Supplementary Materials

The following are available online at https://www.mdpi.com/2072-666X/11/6/581/s1, Figure S1: Procedure of isolating of single NW for an I–V curve measurement.; Figure S2: Force-distance curve of GaAs NW load by a cantilever.

## References

[1] LaPierre R., Chia A., Gibson S., Haapamaki C., Boulanger J., Yee R., Kuyanov P., Zhang J., Tajik N., Jewell N. (2013). III–V nanowire photovoltaics: Review of design for high efficiency. Phys. Status Solidi (RRL)–Rapid Res. Lett..

[2] Green M.A. (2001). Third generation photovoltaics: Ultra-high conversion efficiency at low cost. Prog. Photovolt. Res. Appl..

[3] LaPierre R. (2011). Numerical model of current-voltage characteristics and efficiency of GaAs nanowire solar cells. J. Appl. Phys..

[4] Bauhuis G., Mulder P., Haverkamp E., Huijben J., Schermer J. (2009). 26.1% thin-film GaAs solar cell using epitaxial lift-off. Sol. Energy Mater. Sol. Cells.

[5] Yoshikawa K., Kawasaki H., Yoshida W., Irie T., Konishi K., Nakano K., Uto T., Adachi D., Kanematsu M., Uzu H. (2017). Silicon heterojunction solar cell with interdigitated back contacts for a photoconversion efficiency over 26%. Nat. Energy.

[6] Dhaka V., Haggren T., Jussila H., Jiang H., Kauppinen E., Huhtio T., Sopanen M., Lipsanen H. (2012). High quality GaAs nanowires grown on glass substrates. Nano Lett..

[7] Garnett E.C., Brongersma M.L., Cui Y., McGehee M.D. (2011). Nanowire solar cells. Annu. Rev. Mater. Res..

[8] Krogstrup P., Jørgensen H.I., Heiss M., Demichel O., Holm J.V., Aagesen M., Nygard J., i Morral A.F. (2013). Single-nanowire solar cells beyond the Shockley–Queisser limit. Nat. Photonics.

[9] Holm J.V., Jørgensen H.I., Krogstrup P., Nygård J., Liu H., Aagesen M. (2013). Surface-passivated GaAsP single-nanowire solar cells exceeding 10% efficiency grown on silicon. Nat. Commun..

[10] Cirlin G., Bouravleuv A., Soshnikov I., Samsonenko Y.B., Dubrovskii V., Arakcheeva E., Tanklevskaya E., Werner P. (2010). Photovoltaic properties of p-doped GaAs nanowire arrays grown on n-type GaAs (111) B substrate. Nanoscale Res. Lett..

[11] Tchernycheva M., Rigutti L., Jacopin G., de Luna Bugallo A., Lavenus P., Julien F., Timofeeva M., Bouravleuv A., Cirlin G., Dhaka V. (2012). Photovoltaic properties of GaAsP core–shell nanowires on Si (001) substrate. Nanotechnology.

[12] Åberg I., Vescovi G., Asoli D., Naseem U., Gilboy J.P., Sundvall C., Dahlgren A., Svensson K.E., Anttu N., Björk M.T. (2015). A GaAs nanowire array solar cell with 15.3% efficiency at 1 sun. IEEE J. Photovolt..

[13] Borgström M.T., Magnusson M.H., Dimroth F., Siefer G., Höhn O., Riel H., Schmid H., Wirths S., Björk M., Åberg I. (2018). Towards nanowire tandem junction solar cells on silicon. IEEE J. Photovolt..

[14] Yao M., Cong S., Arab S., Huang N., Povinelli M.L., Cronin S.B., Dapkus P.D., Zhou C. (2015). Tandem solar cells using GaAs nanowires on Si: Design, fabrication, and observation of voltage addition. Nano Lett..

[15] LaPierre R. (2011). Theoretical conversion efficiency of a two-junction III-V nanowire on Si solar cell. J. Appl. Phys..

[16] Hu Y., Chang Y., Fei P., Snyder R.L., Wang Z.L. (2010). Designing the electric transport characteristics of ZnO micro/nanowire devices by coupling piezoelectric and photoexcitation effects. ACS Nano.

[17] Yang Q., Guo X., Wang W., Zhang Y., Xu S., Lien D.H., Wang Z.L. (2010). Enhancing sensitivity of a single ZnO micro-/nanowire photodetector by piezo-phototronic effect. ACS Nano.

[18] Arlt G., Quadflieg P. (1968). Piezoelectricity in iii-v compounds with a phenomenological analysis of the piezoelectric effect. Phys. Status Solidi.

[19] Smith D. (1986). Strain-generated electric fields in [111] growth axis strained-layer superlattices. Solid State Commun..

[20] Boxberg F., Søndergaard N., Xu H. (2012). Elastic and piezoelectric properties of zincblende and wurtzite crystalline nanowire heterostructures. Adv. Mater..

[21] Boxberg F., Søndergaard N., Xu H. (2010). Photovoltaics with piezoelectric core-shell nanowires. Nano Lett..

[22] Chung K.-W., Wang Z., Costa J., Williamson F., Ruden P., Nathan M. (1991). Barrier height change in GaAs Schottky diodes induced by piezoelectric effect. Appl. Phys. Lett..

[23] Calahorra Y., Husmann A., Bourdelain A., Kim W., Vukajlovic-Plestina J., Boughey C., Jing Q., i Morral A.F., Kar-Narayan S. (2019). Highly sensitive piezotronic pressure sensors based on undoped GaAs nanowire ensembles. J. Phys. D Appl. Phys..

[24] Zhang X., Dubrovskii V.G., Sibirev N.V., Ren X. (2011). Analytical study of elastic relaxation and plastic deformation in nanostructures on lattice mismatched substrates. Cryst. Growth Des..

[25] Hübner K. (1973). Piezoelectricity in zincblende-and wurtzite-type crystals. Phys. Status Solidi.

[26] Qiao S., Liu J., Fu G., Ren K., Li Z., Wang S., Pan C. (2018). ZnO nanowire based CIGS solar cell and its efficiency enhancement by the piezo-phototronic effect. Nano Energy.

[27] Pan C., Niu S., Ding Y., Dong L., Yu R., Liu Y., Zhu G., Wang Z.L. (2012). Enhanced Cu_2_S/CdS coaxial nanowire solar cells by piezo-phototronic effect. Nano Lett..

[28] Jiang C., Jing L., Huang X., Liu M., Du C., Liu T., Pu X., Hu W., Wang Z.L. (2017). Enhanced solar cell conversion efficiency of InGaN/GaN multiple quantum wells by piezo-phototronic effect. ACS Nano.

[29] Signorello G., Lörtscher E., Khomyakov P., Karg S., Dheeraj D., Gotsmann B., Weman H., Riel H. (2014). Inducing a direct-to-pseudodirect bandgap transition in wurtzite GaAs nanowires with uniaxial stress. Nat. Commun..

[30] Alekseev P.A., Sharov V.A., Geydt P., Dunaevskiy M.S., Lysak V.V., Cirlin G.E., Reznik R.R., Khrebtov A.I., Soshnikov I.P., Lähderanta E. (2018). Piezoelectric current generation in wurtzite GaAs nanowires. Phys. Status Solidi (RRL)–Rapid Res. Lett..

[31] Bouravleuv A., Ilkiv I., Reznik R., Kotlyar K., Soshnikov I., Cirlin G., Brunkov P., Kirilenko D., Bondarenko L., Nepomnyaschiy A. (2017). New method for MBE growth of GaAs nanowires on silicon using colloidal Au nanoparticles. Nanotechnology.

[32] Harmand J.-C., Patriarche G., Glas F., Panciera F., Florea I., Maurice J.-L., Travers L., Ollivier Y. (2018). Atomic step flow on a nanofacet. Phys. Rev. Lett..

[33] Biermanns A., Breuer S., Trampert A., Davydok A., Geelhaar L., Pietsch U. (2012). Strain accommodation in Ga-assisted GaAs nanowires grown on silicon (111). Nanotechnology.

[34] Mikulik D., Ricci M., Tutuncuoglu G., Matteini F., Vukajlovic J., Vulic N., Alarcon-Llado E., i Morral A.F. (2017). Conductive-probe atomic force microscopy as a characterization tool for nanowire-based solar cells. Nano Energy.

[35] Alekseev P., Dunaevskiy M., Mikhailov A., Lebedev S., Lebedev A., Ilkiv I., Khrebtov A., Bouravleuv A., Cirlin G. (2018). Electrical properties of GaAs nanowires grown on Graphene/SiC hybrid substrates. Semiconductors.

[36] Brantley W. (1973). Calculated elastic constants for stress problems associated with semiconductor devices. J. Appl. Phys..

[37] Hsin C.-L., Mai W., Gu Y., Gao Y., Huang C.-T., Liu Y., Chen L.-J., Wang Z.-L. (2008). Elastic properties and buckling of silicon nanowires. Adv. Mater..

[38] Wang Y.-B., Wang L.-F., Joyce H.J., Gao Q., Liao X.-Z., Mai Y.-W., Tan H.H., Zou J., Ringer S.P., Gao H.-J. (2011). Super deformability and Young’s modulus of GaAs nanowires. Adv. Mater..

[39] Silvaco Atlas. https://www.silvaco.com/products/tcad/device_simulation/atlas/atlas.html.

[40] Alekseev P., Geydt P., Dunaevskiy M., Lähderanta E., Haggrén T., Kakko J.-P., Lipsanen H. (2017). I–V curve hysteresis induced by gate-free charging of GaAs nanowires’ surface oxide. Appl. Phys. Lett..

[41] Lin A., Shapiro J.N., Senanayake P.N., Scofield A.C., Wong P.-S., Liang B., Huffaker D.L. (2012). Extracting transport parameters in GaAs nanopillars grown by selective-area epitaxy. Nanotechnology.

[42] Signorello G., Karg S., Björk M.T., Gotsmann B., Riel H. (2013). Tuning the light emission from GaAs nanowires over 290 meV with uniaxial strain. Nano Lett..

[43] Spicer W., Lindau I., Skeath P., Su C., Chye P. (1980). Unified mechanism for Schottky-barrier formation and III-V oxide interface states. Phys. Rev. Lett..

[44] Freeouf J., Woodall J. (1981). Schottky barriers: An effective work function model. Appl. Phys. Lett..

[45] Alekseev P.A., Dunaevskiy M.S., Cirlin G.E., Reznik R.R., Smirnov A.N., Kirilenko D.A., Davydov V.Y., Berkovits V.L. (2018). Unified mechanism of the surface Fermi level pinning in III-As nanowires. Nanotechnology.

[46] Alekseev P.A., Sharov V.A., Dunaevskiy M.S., Kirilenko D.A., Ilkiv I.V., Reznik R.R., Cirlin G.E., Berkovits V.L. (2019). Control of conductivity of in x Ga1–x as nanowires by applied tension and surface states. Nano Lett..

[47] Łagowski J., Baltov I., Gatos H.C. (1973). Surface photovoltage spectroscopy and surface piezoelectric effect in GaAs. Surf. Sci..

[48] Ibbetson J.P., Fini P., Ness K., DenBaars S., Speck J., Mishra U. (2000). Polarization effects, surface states, and the source of electrons in AlGaN/GaN heterostructure field effect transistors. Appl. Phys. Lett..

[49] Li S., Ware M., Wu J., Minor P., Wang Z., Wu Z., Jiang Y., Salamo G.J. (2012). Polarization induced pn-junction without dopant in graded AlGaN coherently strained on GaN. Appl. Phys. Lett..

[50] Zhang Z., Yao K., Liu Y., Jin C., Liang X., Chen Q., Peng L.-M. (2007). Quantitative analysis of current–voltage characteristics of semiconducting nanowires: Decoupling of contact effects. Adv. Funct. Mater..

[51] Geydt P., Alekseev P., Dunaevskiy M., Lähderanta E., Haggren T., Kakko J.-P., Lipsanen H. (2015). Observation of linear IV curves on vertical GaAs nanowires with atomic force microscope. J. Phys. Conf. Ser..

[52] Suyatin D.B., Jain V., Nebol’sin V.A., Trägårdh J., Messing M.E., Wagner J.B., Persson O., Timm R., Mikkelsen A., Maximov I. (2014). Strong Schottky barrier reduction at Au-catalyst/GaAs-nanowire interfaces by electric dipole formation and Fermi-level unpinning. Nat. Commun..

[53] Zamani M., Tütüncüoglu G., Martí-Sánchez S., Francaviglia L., Güniat L., Ghisalberti L., Potts H., Friedl M., Markov E., Kim W. (2018). Optimizing the yield of A-polar GaAs nanowires to achieve defect-free zinc blende structure and enhanced optical functionality. Nanoscale.

[54] Balaghi L., Bussone G., Grifone R., Hübner R., Grenzer J., Ghorbani-Asl M., Krasheninnikov A.V., Schneider H., Helm M., Dimakis E. (2019). Widely tunable GaAs bandgap via strain engineering in core/shell nanowires with large lattice mismatch. Nat. Commun..

