# Supplementary material for: Effect of the Uniaxial Compression on the GaAs Nanowire Solar Cell
Source: Micromachines (Basel). 2020 Jun 10;11(6):581. doi: 10.3390/mi11060581 (PMC7345117; doi:10.3390/mi11060581)
Supplement: Supplementary file 1 [file micromachines-11-00581-s001.pdf]

Supplementary Material for

Effect of the uniaxial compression on the GaAs nanowire solar cell

Prokhor A. Alekseev[1,*], Vladislav A. Sharov[1,2], Bogdan R. Borodin[1], Mikhail S. Dunaevskiy[1], Rodion R. Reznik[3], and George E. Cirlin[2,4]

[1] Ioffe Institute, Saint-Petersburg, 194021, Russia
[2] Alferov University, Saint-Petersburg 194021, Russia
[3] ITMO University, Saint-Petersburg 197101, Russia
[4] Saint Petersburg Electrotechnical University "LETI", Saint-Petersburg 197376, Russia

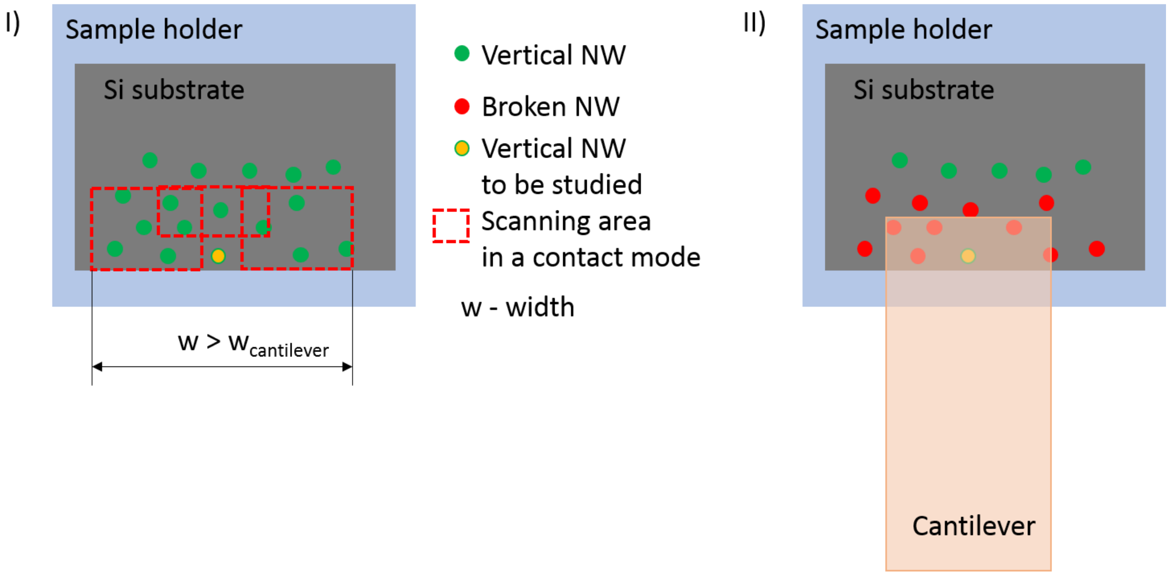

Figure S1. Procedure of isolating of single NW for an I-V curve measurement (top view). The breakdown of neighboring nanowires was carried out as follows: 1) Choosing of a region with NWs near the edge of a Si substrate (at a distance not greater than the length of the cantilever). 2) Obtaining of an AFM image of the region in a semicontact (tapping) nondestructive mode. 3) Scanning in a contact (destructive) mode of areas shown by red dashed rectangles and leading to the NW break (red circles). 4) Studying of an isolated NW (yellow circle) by upside downed cantilever.

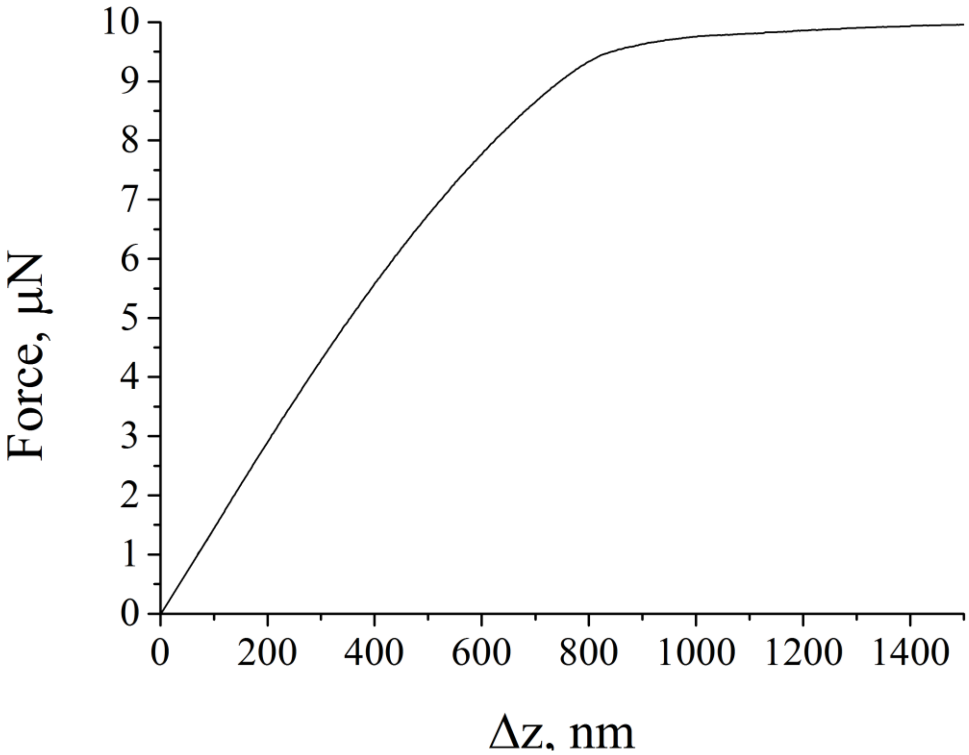

Figure S2. Force-distance curve of GaAs NW load by a cantilever.
